# The Combination of Chinese Medicine Monomers Reduces 7S Globulin-Induced Damage to IPEC-J2 Cells In Vitro by Modulating NF-κB/MAPK Pathway-Related Markers

**DOI:** 10.3390/vetsci13080758

**Published:** 2026-07-30

**Authors:** Ya Wang, Zhengyi Shen, Ling Lv, Zhiguo Li, Chen Yu, Renwu Zhou, Yunhao Su, Youtian Deng, Junliang Deng, Huidan Deng

**Affiliations:** College of Veterinary Medicine, Sichuan Agricultural University, Chengdu 611130, China

**Keywords:** piglet intestinal epithelial cells, soybean 7S globulin, monomer combination of traditional Chinese medicine, inflammatory injury, NF-κB/MAPK pathway

## Abstract

This study aimed to explore the protective effects of different concentration combinations of four traditional Chinese medicine (TCM) monomers (Eleutheroside E, Anemoside B4, Forsythoside A, and Esculin) on porcine intestinal epithelial cell (IPEC-J2) damage induced by soybean 7S globulin, providing preliminary in vitro evidence for future studies on dietary-allergen-induced intestinal injury in piglets. An L_9_ (3^4^) orthogonal experimental design was implemented to identify the optimized ratio of these monomers. IPEC-J2 cells were co-cultured with 5 mg/mL 7S globulin and the selected combinations for 24 h. Cell viability was determined via Cell Counting Kit-8 (CCK-8) assay, while cytokine secretion, oxidative status, and mechanical barrier markers were assessed using enzyme-linked immunosorbent assay (ELISA) and RT-qPCR. The optimal combination was determined to consist of Eleutheroside E at 50 mg/L, Anemoside B4 at 25 mg/L, Forsythoside A at 80 mg/L, and Esculin at 20 mg/L. This formulation significantly alleviated inflammatory damage, upregulated tight-junction-related mRNA expression, and reversed the decrease in cellular viability caused by 7S globulin. Furthermore, Western blot and transcript analyses suggested that this combination exerted its protective effects by modulating the nuclear factor-kappa B/mitogen-activated protein kinase (NF-κB/MAPK) pathway-related markers, reducing the expression of p38 MAPK, c-Jun N-terminal kinase (JNK), NF-κB p65, and inducible nitric oxide synthase (iNOS). In conclusion, this optimized combination of TCM monomers successfully alleviates both inflammatory injury and oxidative stress in IPEC-J2 cells in vitro and holds potential as a promising dietary additive candidate for mitigating dietary-allergen-induced enteropathy in piglets.

## Simple Summary

Soybean 7S globulin, also known as β-conglycinin, is a major dietary storage protein that frequently causes severe diarrhea in weaned piglets, leading to compromised intestinal epithelial damage, acute inflammatory responses, and impaired nutrient absorption. To identify a safe, highly effective, and antibiotic-free intervention, this study systematically evaluated various optimized combinations of four bioactive phytochemicals derived from traditional Chinese medicine—Eleutheroside E, Anemoside B4, Forsythoside A, and Esculin. Our results identified an optimal formulation in which these four monomers, combined at specific ratios, effectively protected porcine intestinal epithelial cells (IPEC-J2) against 7S globulin-induced injury in vitro. This combination attenuated inflammatory and oxidative responses, improved cell viability, and upregulated tight-junction-related mRNA expression in vitro. These effects were associated with changes in NF-κB/MAPK pathway-related markers. These findings provide preliminary in vitro evidence that this optimized natural compound blend may be a potential dietary additive candidate for further evaluation in piglet models.

## 1. Introduction

Soybean and its by-products are extensively used as primary protein sources in modern intensive animal husbandry. However, diets containing high concentrations of soy protein are widely recognized as a principal contributor to hypersensitivity, transient diarrhea, and weight loss in early-weaned piglets [1]. Previous studies have demonstrated that soybeans contain a variety of anti-nutritional factors and allergens, among which soybean 7S globulin (also termed β-conglycinin) is characterized as a major immunogenic allergen. With this soy allergen, direct-contact exposure induces severe intestinal epithelial cell damage and tight junction disintegration, primarily via the activation of the NF-κB/MAPK signaling cascade [2]. Under physiological stress, activation of the NF-κB pathway triggers a major downstream release of pro-inflammatory cytokines [3,4,5,6,7], while the concomitant activation of the JNK and p38 MAPK pathways triggers apoptosis, severe cellular oxidative stress [7,8], and structural barrier compromise [9,10,11].

Most studies indicate that the allergenic properties of 7S globulin are difficult to eliminate [8,12,13]. Consequently, antibiotics have been widely employed to manage diarrhea in weaned piglets. However, antibiotics may cause toxic side effects such as acute allergic reactions and even resistance [14,15,16]. In this context, traditional Chinese medicine (TCM) has emerged as a compelling alternative to conventional antibiotics, reflecting the global transition toward antibiotic-free (ABF) practices in livestock production. Conceptually, TCM is defined as a diverse class of natural medicinal substances—principally of botanical, animal, or mineral origin—that are harvested, undergo special thermal or physical processing, and are administered under the holistic guidance of traditional Chinese medical theories to restore physiological harmony [17]. On one hand, TCM confers multifunctional benefits, encompassing growth promotion, enhanced disease resistance, and immunomodulation in piglets, while presenting the ability to reduce antibiotic resistance. On the other hand, TCM exhibits favorable safety profiles, characterized by low toxicity and negligible tissue residues in food-producing animals [18,19]. Accumulating evidence indicates that traditional Chinese medicine (TCM) monomers, such as Eleutheroside E, Anemoside B4, Forsythoside A, and Esculin, exert multifaceted biological effects, including the promotion of intestinal epithelial cell proliferation and differentiation, immunomodulation, anti-inflammation, antioxidation, and anti-apoptosis [20,21,22,23,24,25,26,27,28,29,30]. Furthermore, our preliminary investigations have substantiated the protective efficacy of these individual herbal extracts against 7S globulin-induced injury in porcine intestinal epithelial cells [31,32].

Eleutheroside E (isolated from *Eleutherococcus senticosus*) is recognized to exert anti-inflammatory activities predominantly through the suppression of pro-inflammatory cytokines and the concomitant promotion of anti-inflammatory IL-10 secretion, which contributes significantly to the protection of intestinal health [28]. Furthermore, it enhances the intestinal mechanical barrier by upregulating the expression of tight junction proteins, thereby preserving the integrity of the intestinal epithelium. Anemoside B4 (the primary active triterpenoid saponin of *Pulsatilla chinensis*) enhances the expression of tight junction-associated proteins, thereby fortifying the intestinal mechanical barrier [22]. Concurrently, it exerts potent anti-inflammatory effects through the modulation of the NF-κB signaling pathway [23]. Forsythoside A (derived from *Forsythia suspensa*) exerts protective effects on the intestinal mucosa, which are primarily attributed to its antioxidant and anti-inflammatory properties, as well as its ability to modulate intestinal immune function [20,26,27]. Esculin (sourced from *Fraxinus chinensis*) attenuates intestinal epithelial cell injury and apoptosis, thereby protecting the intestinal mucosa [24,25]. This protective effect is mediated by reducing the generation of peroxidation products, enhancing the body’s antioxidant capacity, modulating NF-κB/MAPK pathway-related markers, and modulating the expression of apoptosis-related genes [30].

Importantly, emerging evidence indicates that combined herbal formulations often yield far superior protective outcomes compared to isolated single-monomer applications, reflecting the multi-component and multi-target characteristics of traditional Chinese medicine formulations [33,34]. To design a highly efficient, targeted green feed candidate for managing soybean allergen-induced enteritis, this study utilized an orthogonal experimental design to optimize a unique combination of Eleutheroside E, Anemoside B4, Forsythoside A, and Esculin. Using the non-transformed porcine intestinal epithelial cell line (IPEC-J2) as an in vitro model, we systematically evaluated whether this optimized monomer formulation could alleviate soybean 7S globulin-induced cell viability loss, acute inflammation, oxidative stress, and mechanical barrier damage, and further explored its regulatory effects on the NF-κB/MAPK signaling pathway. Ultimately, this study aims to provide initial in vitro evidence and theoretical references for developing novel phytochemical interventions against piglet post-weaning diarrhea.

## 2. Materials and Methods

### 2.1. Materials

Soybean 7S globulin (purity: 83 ± 3%, lot no.: 20211118) was purchased from Huaibei Fudelaibo Food Technology Co., Ltd., Huaibei, China. The intestinal porcine epithelial cell line (IPEC-J2), which is a non-transformed, non-tumorigenic cell line originally isolated from the jejunal epithelium of a neonatal unsuckled piglet in 1989, was obtained from Shanghai Yingwan Biotechnology Co., Ltd., Shanghai, China. To maintain the high reproducibility of the epithelial physical barrier and metabolic activity, IPEC-J2 cells between passages 75 and 85 were utilized in this study. Four traditional Chinese medicine (TCM) monomers were selected for this study: Eleutheroside E (Purity ≥ 98%, Cat. No.: B20572), Anemoside B4 (Purity ≥ 98%, Cat. No.: B20529), Forsythoside A (Purity ≥ 98%, Cat. No.: B20727), and Esculin (Purity ≥ 98%, Cat. No.: B20989). All monomers were supplied by Shanghai Yuanye Biotechnology Co., Ltd., Shanghai, China.

### 2.2. Orthogonal Experimental Design

The selection of the baseline concentrations for the individual TCM monomers in this study was strictly grounded in prior systematic single-factor screening experiments conducted by our research group under identical in vitro conditions (IPEC-J2 cells challenged with 5 mg/mL soybean 7S globulin). Specifically, Fu evaluated the protective and anti-apoptotic properties of Esculin over a wide concentration range (1.25 to 50 mg/L) and demonstrated that 20 mg/L represented the peak active concentration that maximized cell viability and minimized lipid peroxidation [31]. Concurrently, Wan screened the individual protective and mechanical barrier-preserving profiles of Eleutheroside E, Anemoside B4, and Forsythoside A, establishing that Eleutheroside E at 50 mg/L, Anemoside B4 at 50 mg/L, and Forsythoside A at 40 mg/L represented the optimal individual concentrations that maximized tight-junction protein expressions and minimized pro-inflammatory cytokine release. Based on these previous single-factor screening data, we chose the optimal single-agent concentration of each monomer—50 mg/L Eleutheroside E, 50 mg/L Anemoside B4, 40 mg/L Forsythoside A, and 20 mg/L Esculin—as our baseline concentrations [32]. To optimize the combination, these baseline concentrations were systematically scaled up (2×) and scaled down (0.5×). A four-factor, three-level L_9_ (3^4^) orthogonal experimental design was implemented according to the parameters specified in Table 1. The higher-dose level was designed to evaluate whether increasing the combined concentrations could further enhance the protective effect or approach a potential cytotoxic threshold. The lower-dose level was designed to determine whether comparable protective effects could be maintained at reduced concentrations. All monomer stock solutions were prepared in dimethyl sulfoxide (DMSO) and subsequently diluted in complete culture medium. The final concentration of DMSO was strictly maintained below 0.1% (*v*/*v*) in all treatment groups, a vehicle concentration previously confirmed to have no cytotoxic or stimulatory effects on IPEC-J2 cell viability [32].

The experimental design generated 10 distinct treatment groups, which included the untreated Control group and 9 combinations (I to IX), as detailed in Table 2. Each treatment group was evaluated with six parallel biological replicates.

### 2.3. Cell Viability Analysis (CCK-8 Assay)

#### 2.3.1. Identification of Combinatorial Formulations Enhancing Cell Viability

IPEC-J2 cells in the logarithmic growth phase were trypsinized, suspended at a density of 1 × 10^5^ cells/mL and seeded into 96-well culture plates (100 μL per well). Following an overnight incubation at 37 °C under 5% CO_2_ to allow cell attachment, the medium was replaced with treatment media containing the orthogonal monomer combinations (Groups I–IX) and cultured for an additional 24 h. Thereafter, 10 μL of CCK-8 assay reagent was added to each well, and the cells were incubated in the dark at 37 °C for 4 h. The optical density (OD) of each well was recorded at 450 nm on a microplate reader to determine the relative survival rate of the cells [6].

#### 2.3.2. Effects of the Identified Combinations on IPEC-J2 Cells Exposed to 7S Globulin

The exposure condition of 5 mg/mL soybean 7S globulin for 24 h was selected to establish the IPEC-J2 cell injury model based mainly on previous work from our research group. Yang et al. reported that treatment with 5 mg/mL soybean 7S globulin for 24 h reduced IPEC-J2 cell viability to approximately 50% and reproducibly induced epithelial barrier dysfunction, as indicated by increased fluorescein sodium permeability, impaired membrane integrity, abnormal cytokine secretion, and downregulated tight-junction protein expression [35]. Therefore, this concentration was considered suitable for inducing moderate and stable in vitro cellular injury while maintaining sufficient viable cells for subsequent protective evaluation. Based on this rationale, IPEC-J2 cells were co-cultured with 5 mg/mL soybean 7S globulin and the selected monomer combinations I, VI, and VIII in 96-well plates for 24 h. After incubation, 10 μL of Cell Counting Kit-8 reagent was added to each well, and the plate was incubated at 37 °C in the dark for 4 h. The optical density at 450 nm was then measured using a microplate reader to calculate the relative viability of IPEC-J2 cells.

### 2.4. Enzyme-Linked Immunosorbent Assay (ELISA)

After culturing the cells as in 2.3.2, collect the cell supernatant, and strictly follow the operating instructions of the ELISA kit (Shanghai Yuanju Biotechnology Center, Shanghai, China) to detect the contents of pro-inflammatory factors IL-6, TNF-α, IL-1β, anti-inflammatory factors IL-4, IL-10, and cellular oxidation indicators SOD, CAT, T-AOC, GSH-Px, and MDA in the supernatant of IPEC-J2 cells.

### 2.5. RT-qPCR Analysis

RNA was extracted from the cells according to the instructions of the Rapid RNA Extraction Kit (Foregene, Chengdu, China). Then, reverse transcription was performed to synthesise cDNA, strictly following the instructions for the Reverse Transcription Kit (Foregene, Chengdu, China). cDNA from the reverse transcription was diluted five-fold, and RT-qPCR was carried out according to the instructions for the Fluorescence Quantitative PCR Kit (Foregene, Chengdu, China). Determination of mRNA expression of Zonula Occludens-1 (*ZO-1*), *Claudin-1*, and *Occludin* tight junction protein genes in IPEC-J2 cells. The mRNA expression of NF-κB/MAPK signaling pathway-related protein genes in IPEC-J2 cells was further determined based on the assay results. The primers were designed and synthesised by Shanghai Sangon Biotech, Shanghai, China (Table 3). According to the 2^−^^∆∆Ct^ method [36], the relative expression of the target genes in different groups of cells was calculated.

### 2.6. Western Blot Analysis

After a period of exposure in the medium, as detailed in Section 2.3.2, the cells were incubated with lysis buffer. Protein concentrations were determined using a BCA Protein Assay Kit (ThermoWaltham, MA, USA). Protein samples were separated by SDS-PAGE (Servicebio, Wuhan, China) and then transferred onto a nitrocellulose membrane. Subsequently, the membranes were blocked with 5% nonfat milk (Solabio, Beijing, China) and then incubated with primary antibodies against *P38 MAPK* (1:1000, Servicebio, Wuhan, China), *JNK* (1:1000, Servicebio, Wuhan, China), *iNOS* (1:1000, Servicebio, Wuhan, China), *NF-κB p65* (1:1000, Servicebio, Wuhan, China), and *β-actin* (1:3000, Servicebio, Wuhan, China) overnight at 4 °C. After washing, membranes were incubated with secondary antibody dilution buffer (1:3000, Servicebio, Wuhan, China) for 1 h at room temperature. Antibody binding was visualized using ECL reagent (Servicebio, Wuhan, China). The relative levels of each protein to β-actin were determined by densitometry analysis with ImageJ software version 1.54p (National Institutes of Health).

### 2.7. Data Analysis and Statistic

All raw experimental data were organized using Excel and statistically analyzed using SPSS 26.0 software. Prior to statistical comparisons, the normality of the data was evaluated using the Shapiro–Wilk test, and the homogeneity of variance was assessed using Levene’s test. For multi-group comparisons, statistical significance was determined using one-way analysis of variance (ANOVA) followed by Tukey’s post hoc test for pairwise multiple comparisons. Quantitative data are expressed as the mean ± standard error of the mean (SEM). Statistical significance was defined at *p* < 0.05, and high significance was defined at *p* < 0.01. All statistical graphs were constructed using GraphPad Prism 11.0.

## 3. Results

### 3.1. Effects of Different Combinations of Herbal Monomers on the Viability of IPEC-J2 Cells

**Figure 1:**
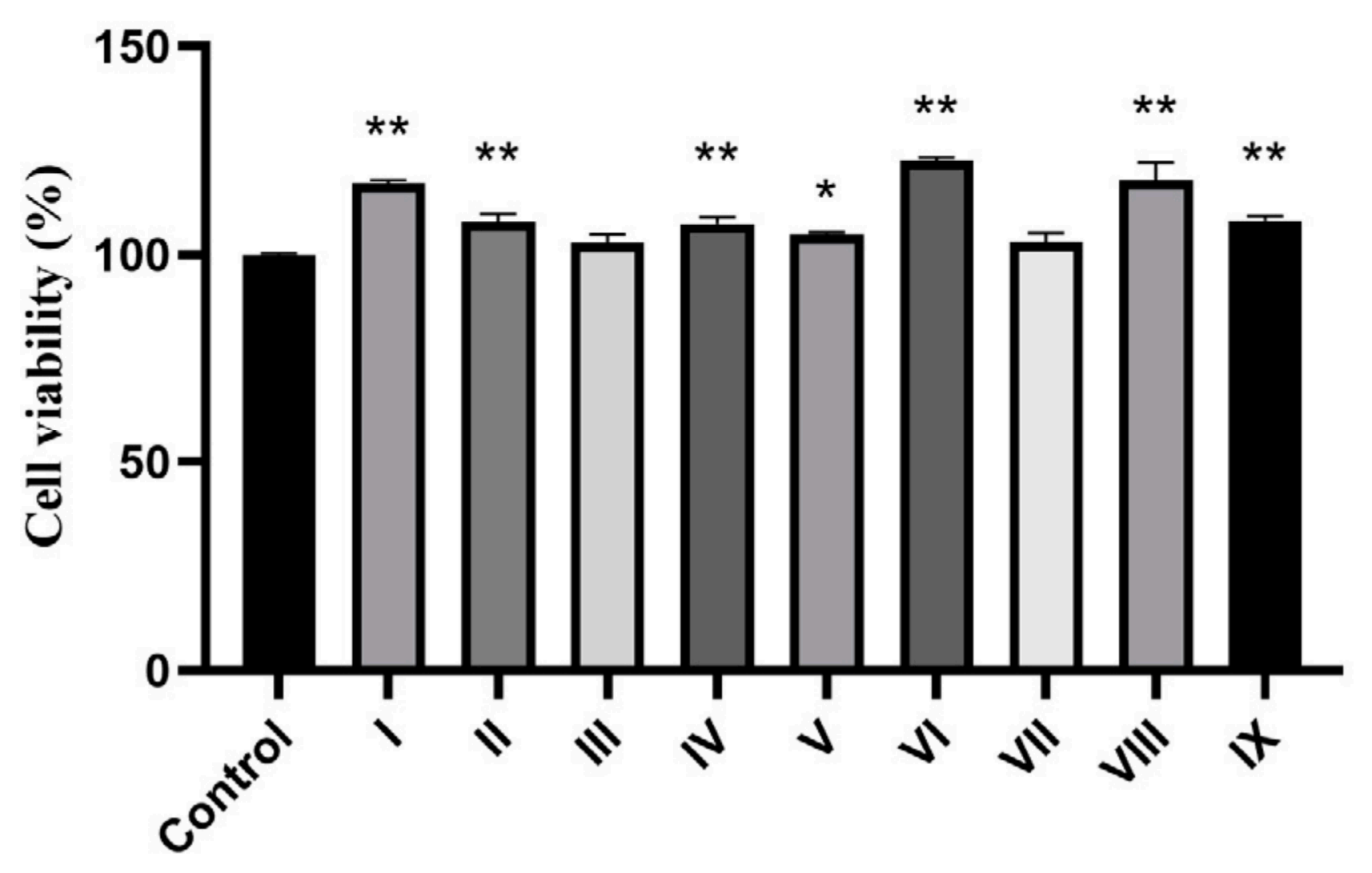
Effects of different combinations of four Chinese medicine monomers on the viability of IPEC-J2 cells. Note: Compared with the control group, * *p* < 0.05 and ** *p* < 0.01. The specific concentrations are shown in Table 2. Data are presented as mean ± SEM (*n* = 6).

The effects of nine combinations of different concentrations of Eleutheroside E (A), Anemoside B4 (B), Forsythoside A (C), and Esculin (D) on the viability of IPEC-J2 cells are shown in Figure 1. Compared with the control group, treatment with combinations I, II, IV, V, VI, VIII, and IX significantly increased the viability of IPEC-J2 cells (*p* < 0.05 or *p* < 0.01). Conversely, combinations III and VII exerted no significant effect on cell viability (*p* > 0.05). To clarify the relative contribution of the four monomers in the orthogonal combinations, range analysis was performed based on the cell viability results of the nine orthogonal combinations. The range analysis in Table 4 showed that the influence order of the four factors was C > A > B > D, indicating that Forsythoside A had the greatest effect on cell viability. The predicted optimal combination was A2B3C1D2, corresponding to Group VI, which consisted of 50 mg/L Eleutheroside E, 25 mg/L Anemoside B4, 80 mg/L Forsythoside A, and 20 mg/L Esculin. Groups I, VI, and VIII showed relatively high cell viability among the nine orthogonal combinations and did not exhibit obvious cytotoxicity; therefore, they were selected as candidate combinations for subsequent evaluation in the 7S globulin-induced injury model. It should be noted that cell viability was used only as a preliminary screening indicator, whereas the final optimal combination was determined based on comprehensive evaluation of inflammatory, oxidative-stress, and tight-junction-related indicators in subsequent experiments.

### 3.2. Effects of Three Combinations of Herbal Monomers on 7S Globulin-Induced Cell Viability

**Figure 2:**
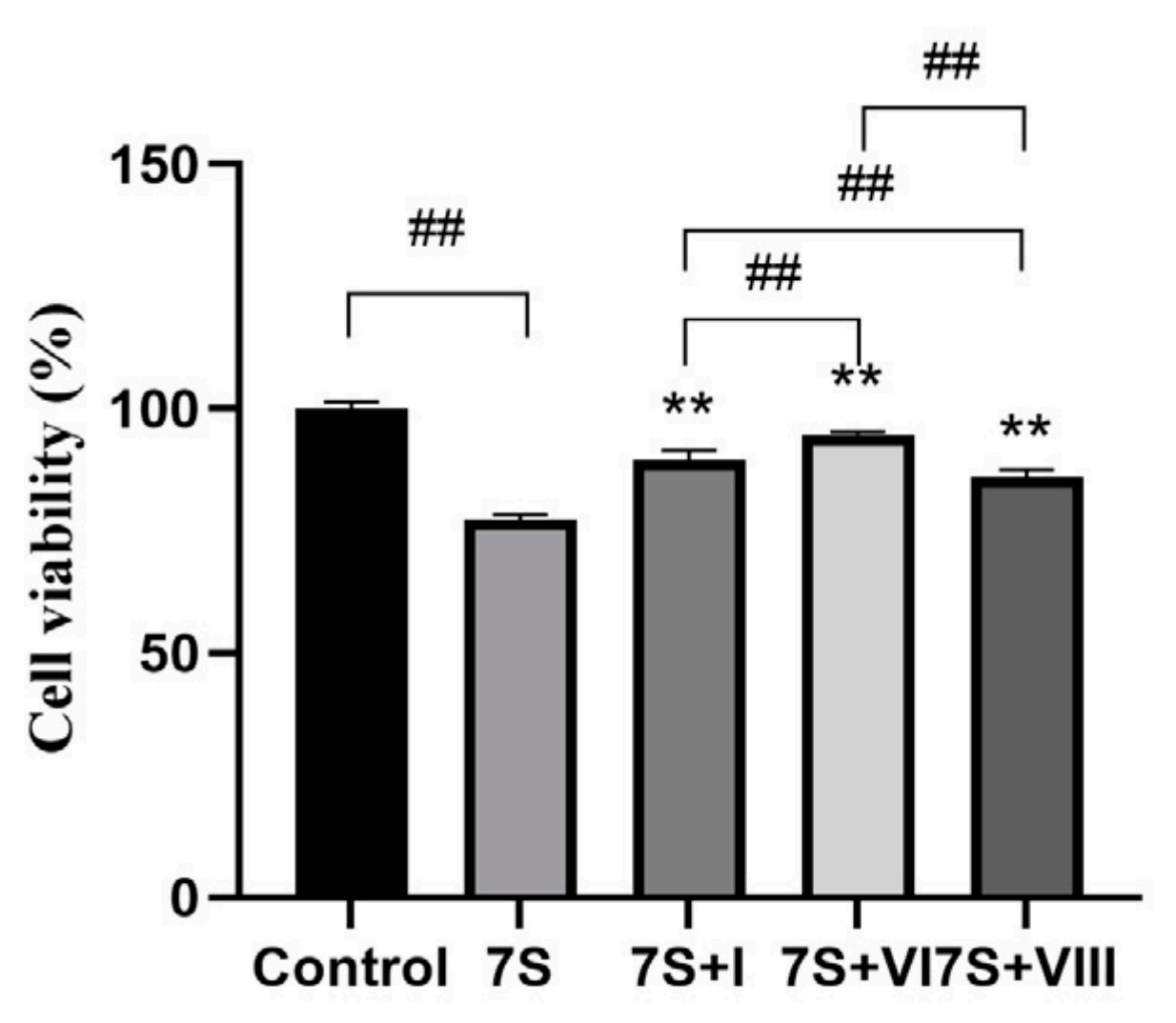
Effect of three combinations of TCM monomers on 7S globulin-induced viability of IPEC-J2 cells. Note: Control, control group; 7S, 7S model group; 7S + I, 7S + combination I; 7S + VI, 7S + combination VI; 7S + VIII, 7S + combination VIII. The specific concentrations are shown in Table 2. Comparison between groups, ## *p* < 0.01, and compared with the 7S model group, ** *p* < 0.01. Data are presented as mean ± SEM (*n* = 6).

The protective effects of the three selected combinations (I, VI, and VIII) on IPEC-J2 cell viability under 7S globulin-induced injury are shown in Figure 2. Exposure of IPEC-J2 cells to 5 mg/mL soybean 7S globulin for 24 h resulted in a highly significant reduction in cell viability compared to the untreated Control group (*p* < 0.01). However, when co-incubated with any of the three selected TCM combinations, cellular viability was significantly restored compared to the 7S globulin model group (*p* < 0.01). Among these rescue groups, the 7S + combination VI group (A 50 mg/L, B 25 mg/L, C 80 mg/L, and D 20 mg/L) exhibited the most prominent protective effect, maintaining the highest survival rate under allergen challenge.

### 3.3. Effects of Three Combinations of Herbal Monomers on 7S Globulin-Induced Pro-Inflammatory Factors in IPEC-J2 Cells

**Figure 3:**
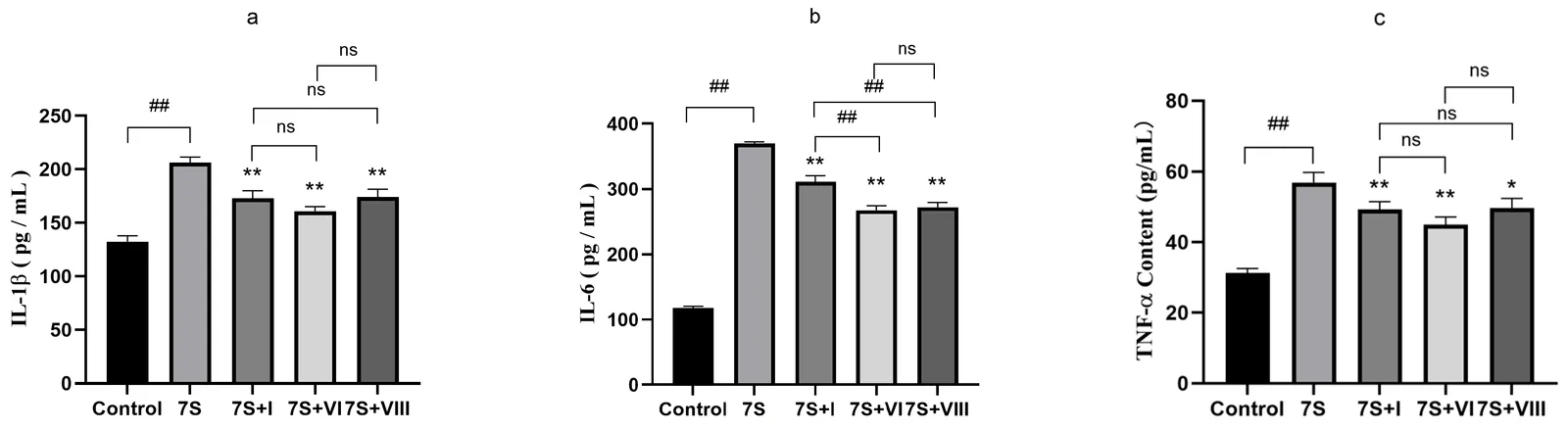
Effect of three combinations of TCM monomers on 7S globulin-induced pro-inflammatory factors IL-1β (**a**), IL-6 (**b**), and TNF-α (**c**) in IPEC-J2 cells. Note: Control, control group; 7S, 7S model group; 7S + I, 7S + combination I; 7S + VI, 7S + combination VI; 7S + VIII, 7S + combination VIII. The specific concentrations are shown in Table 2. Comparison between groups, ## *p* < 0.01, compared with the 7S model group, * *p* < 0.05, ** *p* < 0.01; ns, not significant. Data are presented as mean ± SEM (*n* = 6).

The regulatory effects of the three selected monomer combinations on 7S globulin-induced inflammatory cytokine secretion are presented in Figure 3. Following 24 h of exposure to 5 mg/mL soybean 7S globulin, the levels of pro-inflammatory factors IL-1β, IL-6, and TNF-α in the cell supernatant were significantly elevated compared with the control group (*p* < 0.01). In contrast, co-treatment with any of the three monomer combinations significantly reduced the levels of IL-1β and IL-6 induced by 7S globulin (*p* < 0.01). Under inter-group comparison, combination VI demonstrated the most pronounced inhibitory effect on IL-6, exhibiting a significantly stronger recovery than combination I (*p* < 0.01). Furthermore, combinations I, VI, and VIII all significantly reduced the TNF-α content in the supernatant of cells challenged with 7S globulin (*p* < 0.05 or *p* < 0.01). The inhibitory effects on IL-1β and TNF-α secretion did not differ significantly among the three formulations (*p* > 0.05).

### 3.4. Effects of Three Combinations of Herbal Monomers on 7S Globulin-Induced Anti-Inflammatory Factors in IPEC-J2 Cells

**Figure 4:**
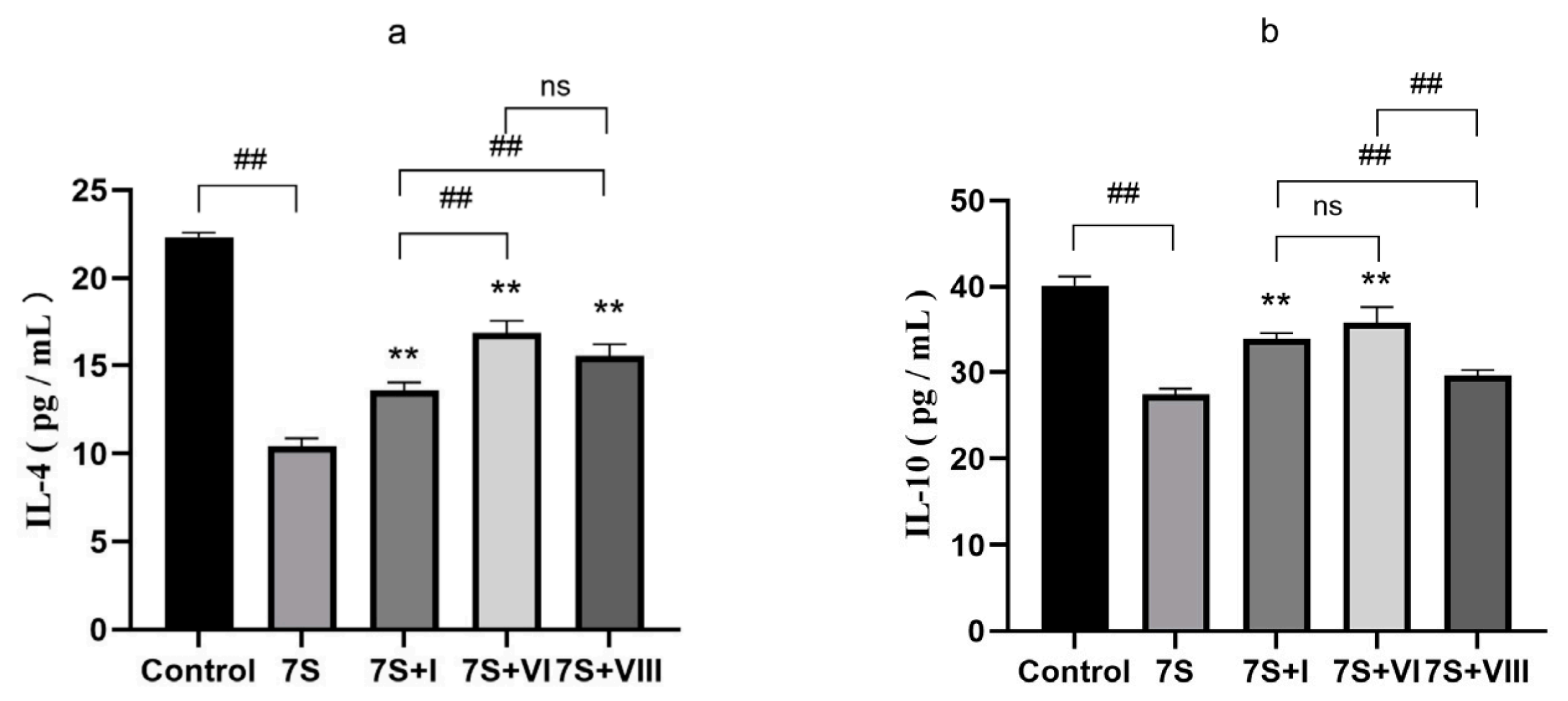
Effect of three combinations of TCM monomers on 7S globulin-induced anti-inflammatory factors IL-4 (**a**) and IL-10 (**b**) in IPEC-J2 cells. Note: Control, control group; 7S, 7S model group; 7S + I, 7S + combination I; 7S + VI, 7S + combination VI; 7S + VIII, 7S + combination VIII. The specific concentrations are shown in Table 2. Comparison between groups, ## *p* < 0.01, compared with the 7S model group, ** *p* < 0.01; ns, not significant. Data are presented as mean ± SEM (*n* = 6).

The protective effects of the monomer combinations on anti-inflammatory cytokine secretion under allergen challenge are shown in Figure 4. Compared with the control group, anti-inflammatory cytokine levels of IL-4 and IL-10 in the cell culture supernatant decreased significantly after 24 h of 5 mg/mL 7S globulin exposure (*p* < 0.01). However, co-incubation with combinations I, VI, and VIII significantly reversed the downregulation of IL-4 (*p* < 0.01). Among these rescue groups, combination VI demonstrated the most pronounced restorative effect on IL-4 levels, which was significantly higher than that of combination I (*p* < 0.01).

Furthermore, combinations I and VI significantly rescued the secretion of IL-10 (*p* < 0.01). Conversely, combination VIII exerted no significant effect on IL-10 recovery (*p* > 0.05). The restorative effect of combination VI on IL-10 levels was significantly greater than that of combination VIII (*p* < 0.01).

### 3.5. Effects of Three Combinations of Herbal Monomers on 7S Globulin-Induced Oxidative Damage in IPEC-J2 Cells

**Figure 5:**
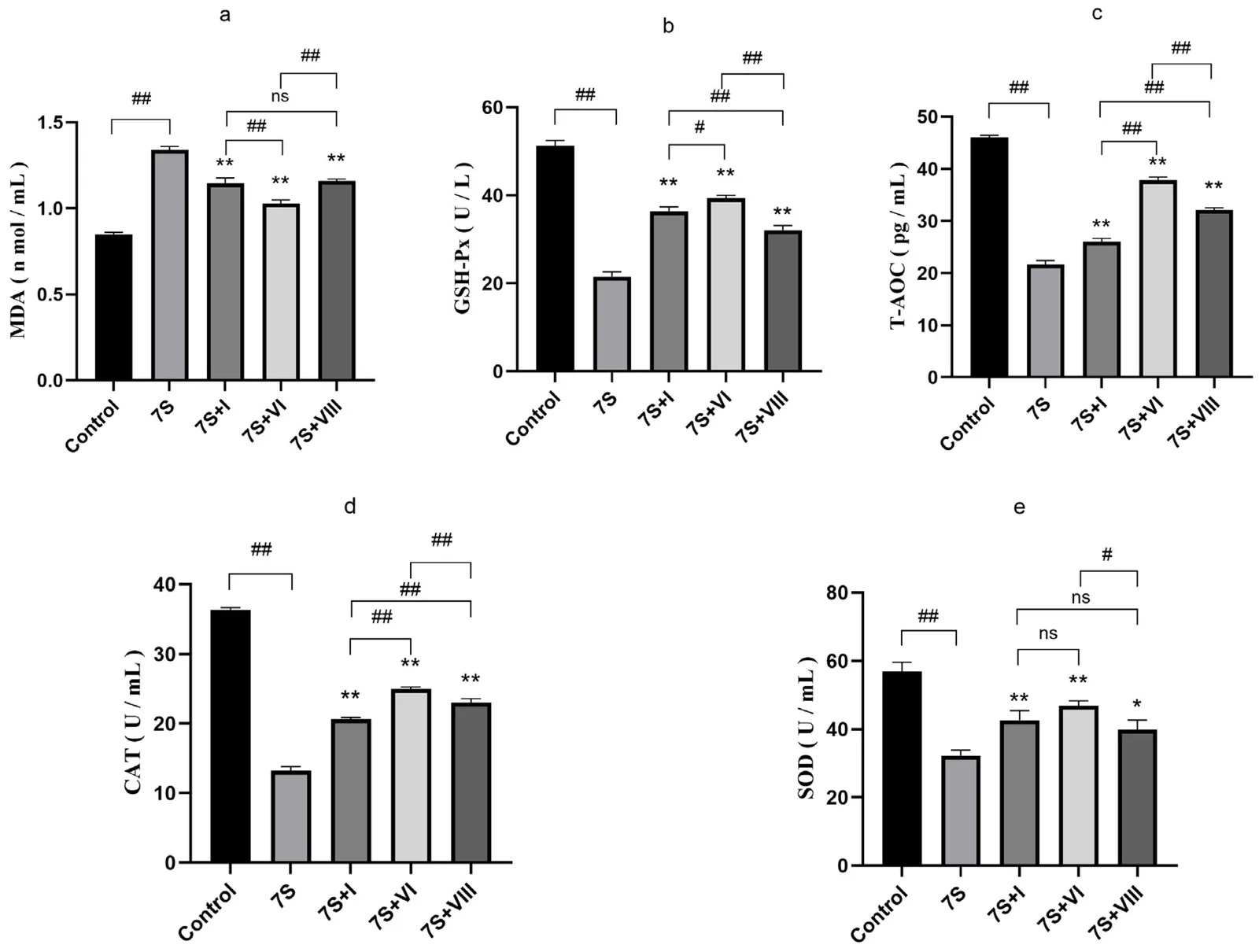
Effects of three combinations of TCM monomers on the MDA (**a**), GSH-Px (**b**), T-AOC (**c**), CAT (**d**), and SOD (**e**) content of IPEC-J2 cells induced by 7S globulin. Note: Control, control group; 7S, 7S model group; 7S + I, 7S + combination I; 7S + VI, 7S + combination VI; 7S + VIII, 7S + combination VIII. The specific concentrations are shown in Table 2. Comparison between groups, # *p* < 0.05, ## *p* < 0.01, compared with the 7S model group, * *p* < 0.05, ** *p* < 0.01; ns, not significant. Data are presented as mean ± SEM (*n* = 6).

The antioxidant efficacy of the three selected combinations on 7S globulin-treated cells is detailed in Figure 5. Compared with the control group, 24 h of 7S globulin challenge induced severe oxidative damage in IPEC-J2 cells, marked by highly significant decreases in intracellular GSH-Px, SOD, CAT, and T-AOC activities alongside a dramatic elevation in lipid peroxidation MDA levels (*p* < 0.01). In contrast, co-treatment with all three monomer combinations significantly reversed these oxidative trends, restoring intracellular T-AOC, CAT, and GSH-Px activities and significantly reducing cellular MDA content (*p* < 0.01). Inter-group analysis demonstrated that combination VI exhibited the most prominent antioxidant profile, outperforming combinations I and VIII (*p* < 0.01). Additionally, combinations I and VI significantly increased cellular SOD activity compared with the 7S model (*p* < 0.01), whereas combination VIII increased SOD activity to a lesser extent (*p* < 0.05). While no significant difference was observed between combinations I and VI in SOD restoration (*p* > 0.05), combination VI was significantly more effective than combination VIII (*p* < 0.05).

### 3.6. Effects of Three Combinations of Herbal Monomers on 7S Globulin-Induced Mechanical Barrier Damage in IPEC-J2 Cells

**Figure 6:**
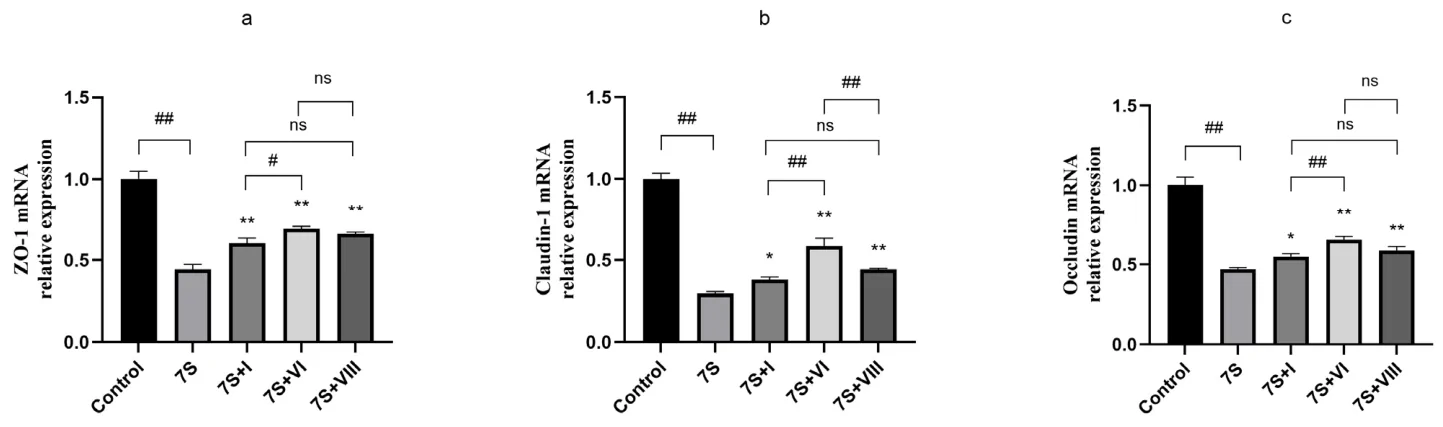
Effect of three combinations of TCM monomers on the mRNA expression of tightly linked ZO-1 (**a**), Claudin-1 (**b**), and Occludin (**c**) in 7S globulin-induced IPEC-J2 cells. Note: Control, control group; 7S, 7S model group; 7S + I, 7S + combination I; 7S + VI, 7S + combination VI; 7S + VIII, 7S + combination VIII. The specific concentrations are shown in Table 2. Comparison between groups, # *p* < 0.05, ## *p* < 0.01; compared with the 7S model group, * *p* < 0.05, ** *p* < 0.01; ns, not significant. Data are presented as mean ± SEM (*n* = 6).

The protective effects of the monomer combinations on the tight junction mechanical barrier of IPEC-J2 cells are shown in Figure 6. Compared with the control group, 24 h of exposure to 7S globulin significantly reduced the relative mRNA transcript levels of the core tight junction proteins *ZO-1*, *Claudin-1*, and *Occludin* (*p* < 0.01). Conversely, co-treatment with combinations I, VI, and VIII significantly rescued *ZO-1* mRNA transcription (*p* < 0.01). Notably, combination VI was significantly more effective than combination I in upregulating *ZO-1* transcription (*p* < 0.05). Furthermore, combinations VI and VIII significantly increased *Occludin* and *Claudin-1* mRNA levels (*p* < 0.01), whereas combination I increased their transcription to a lesser extent (*p* < 0.05). Under inter-group comparison, combination VI was significantly better than combinations I and VIII in increasing the mRNA expression of *Occludin* (*p* < 0.01). There was no significant difference between combinations VI and VIII in upregulating *Claudin-1* mRNA transcription (*p* > 0.05), but combination VI was significantly superior to combination I (*p* < 0.05).

### 3.7. Effects of Optimal Combinations on the 7S Globulin-Induced NF-κB/MAPK Signalling Pathway in IPEC-J2 Cells

**Figure 7:**
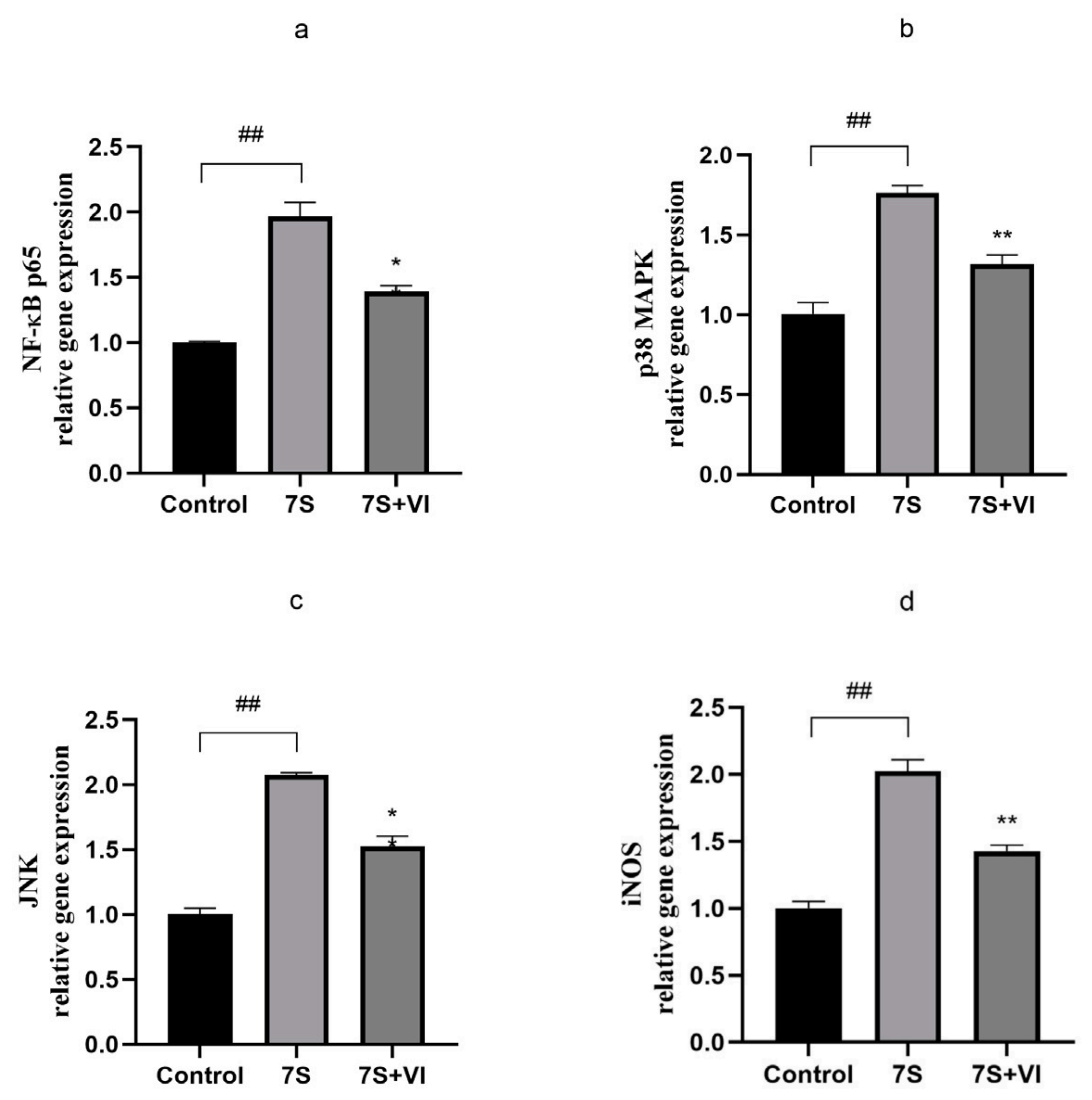
The effect of the optimal combination on the mRNA expression of NF-κB/MAPK signaling pathway-related proteins *NF-κB p65* (**a**), *P38 MAPK* (**b**), *JNK* (**c**), and *iNOS* (**d**) in IPEC-J2 cells induced by 7S globulin. Note: Control, control group; 7S, 7S model group; 7S + VI, 7S + combination VI. The specific concentrations are shown in Table 2. Comparison between groups, ## *p* < 0.01; compared with the 7S model group, * *p* < 0.05, ** *p* < 0.01. Data are presented as mean ± SEM (*n* = 6).

**Figure 8:**
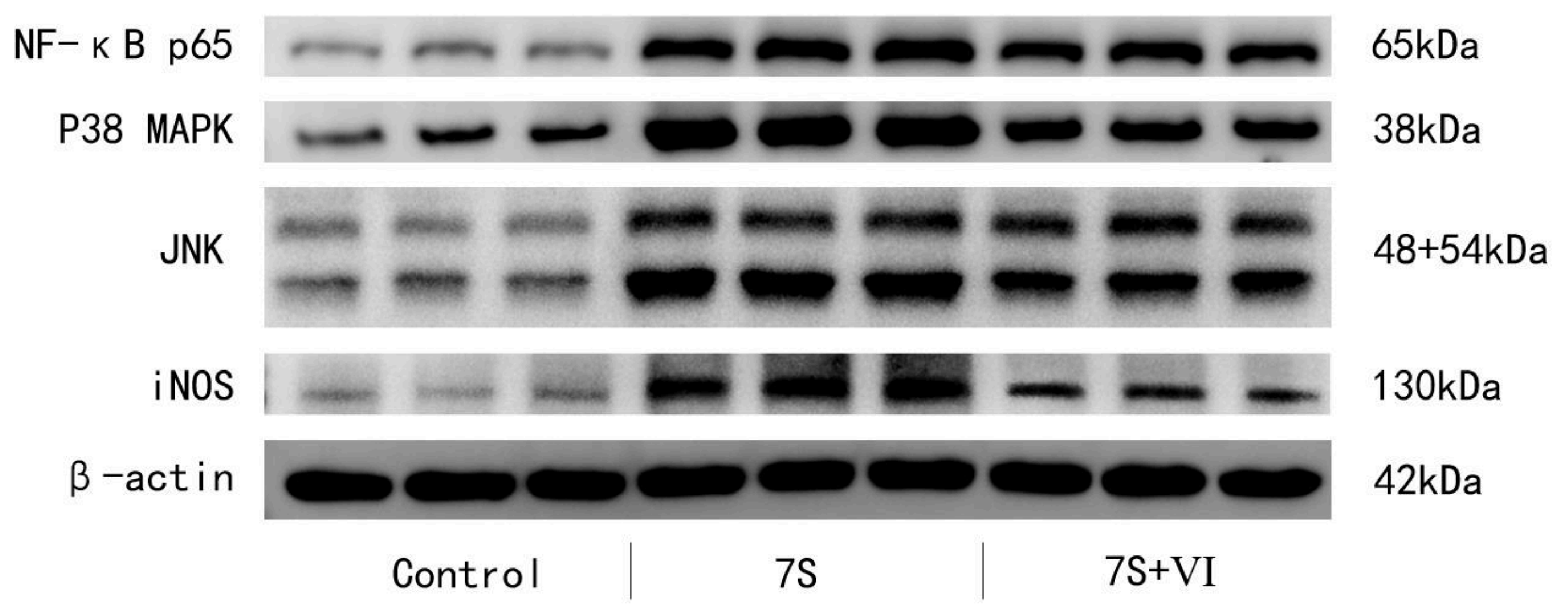

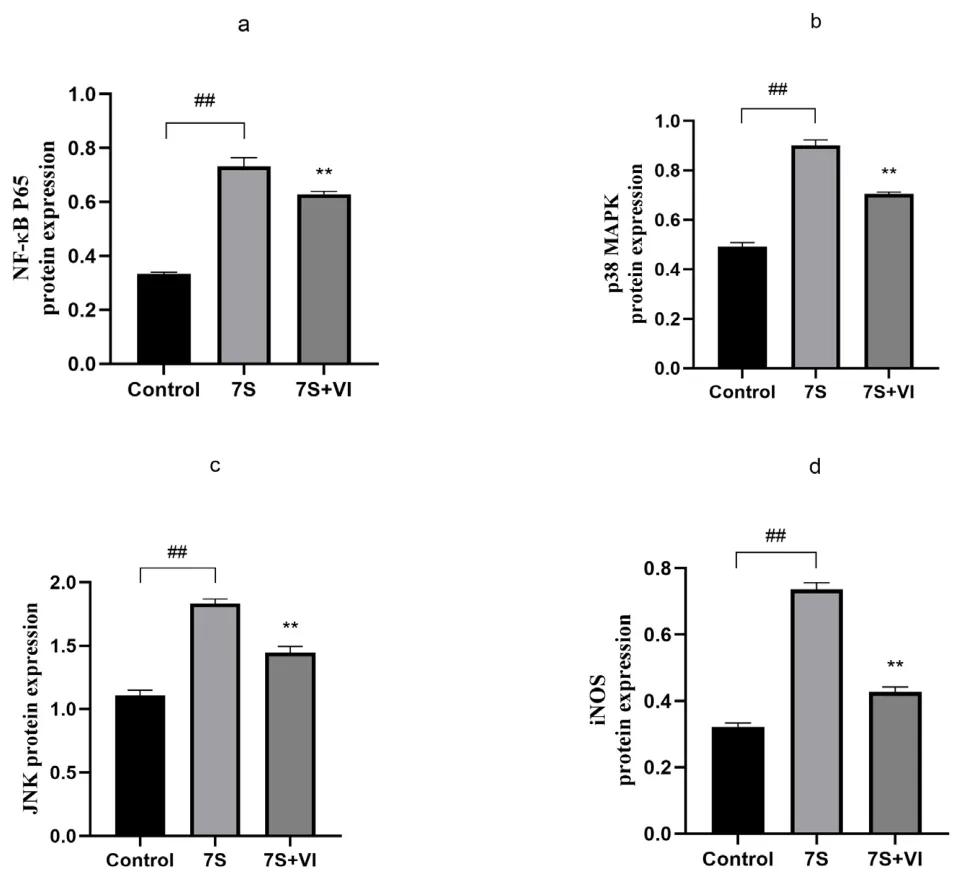
Effect of the optimal combination on the expression of NF-κB/MAPK signaling pathway-related proteins NF-κB p65 (**a**), P38 MAPK (**b**), JNK (**c**), and iNOS (**d**) in IPEC-J2 cells induced by 7S globulin. Note: Control, control group; 7S, 7S model group; 7S + VI, 7S + combination VI. The specific concentrations are shown in Table 2. Comparison between groups, ## *p* < 0.01; compared with the 7S model group, ** *p* < 0.01. Data are presented as mean ± SEM (*n* = 6). The corresponding original uncropped Western blot images and densitometric data are shown in Figures S1–S5 and Table S1.

Based on its outstanding performance across all endpoints, combination VI (50 mg/L Eleutheroside E, 25 mg/L Anemoside B4, 80 mg/L Forsythoside A, and 20 mg/L Esculin) was selected as the optimized combination. The regulatory effects of this formulation on transcription and protein expression within the inflammatory NF-κB/MAPK/iNOS cascade are presented in Figure 7 and Figure 8. RT-qPCR analysis showed that exposure to 5 mg/mL soybean 7S globulin alone for 24 h significantly increased the relative mRNA transcript levels of the key inflammatory signaling genes *NF-κB p65*, *p38 MAPK,* *JNK*, and the downstream enzyme *iNOS* compared with the control group (*p* < 0.01). Conversely, co-treatment with the optimal monomer combination (7S + combination VI) significantly suppressed the relative mRNA expression of *NF-κB p65*, *p38 MAPK*, *JNK*, and *iNOS* (*p* < 0.01), returning their transcription toward baseline levels. This transcriptional regulation was further confirmed at the translational level by Western blot analysis. Exposure to 7S globulin alone significantly increased the protein expression levels of NF-κB p65, p38 MAPK, JNK, and iNOS in IPEC-J2 cells compared with the control group (*p* < 0.01). In contrast, co-incubation with the optimal combination for 24 h significantly blocked these increases, causing a highly significant reduction in the protein levels of NF-κB p65, p38 MAPK, JNK, and iNOS (*p* < 0.01) compared with the 7S globulin model group.

## 4. Discussion

Diarrheal disease triggered by dietary soybean proteins remains a major concern in the swine industry, leading to compromised gut health and high mortality in newly weaned piglets. Early-weaned piglets suffer from a physiological insufficiency of digestive enzymes, which prevents the effective hydrolysis of intact feed proteins. Consequently, immunogenic storage globulins, such as soybean 7S globulin, penetrate the mucosal barrier intact. These proteins bind to local immune and epithelial receptors, triggering severe mucosal inflammation, tight junction breakdown, paracellular leakage, and nutrient malabsorption [37,38,39]. Our study demonstrated that an optimized combination of four TCM-derived monomers—Eleutheroside E, Anemoside B4, Forsythoside A, and Esculin—effectively alleviated 7S globulin-induced inflammatory injury, oxidative stress, and mechanical barrier damage in porcine IPEC-J2 epithelial cells.

In this study, an L_9_(3^4^) orthogonal experimental design was used to optimize the concentration combination of the four monomers and identify an effective compound formulation. Traditional single-factor screening designs evaluate individual parameters independently, which overlooks the complex, multi-target pharmacodynamic interactions that occur when multiple bioactive components are combined. Through systematic analysis and range optimization based on our prior single-factor concentration range [31,32], the optimal optimized ratio was identified as combination VI, consisting of 50 mg/L Eleutheroside E, 25 mg/L Anemoside B4, 80 mg/L Forsythoside A, and 20 mg/L Esculin. Under allergen challenge, this optimized blend significantly restored IPEC-J2 cell viability, outperforming the individual monomers and alternative orthogonal combinations.

This favorable protective effect may be attributed to the multi-component and multi-target characteristics of the optimized formulation, which simultaneously modulates several key processes involved in soy-protein-induced epithelial injury [40,41,42,43]. First, we observed a dramatic reduction in key pro-inflammatory cytokines (IL-1β, IL-6, and TNF-α) alongside a highly significant recovery of anti-inflammatory markers (IL-4 and IL-10) in the cell supernatant. Under pathological challenge, unchecked pro-inflammatory cytokine release damages cellular systems, while anti-inflammatory cytokines are critical for restoring mucosal homeostasis. The observed shifts in the cytokine profile suggest that the monomer combination effectively dampens the hyper-inflammatory state triggered by 7S globulin.

Second, the optimized combination significantly enhanced the cellular enzymatic antioxidant defense system. Under 7S globulin challenge, cells exhibited a sharp drop in SOD, CAT, GSH-Px, and T-AOC activities alongside a substantial accumulation of MDA, indicating severe lipid peroxidation and membrane disruption. Elevated intracellular reactive oxygen species (ROS) oxidize membrane lipids and structural proteins, compromising cellular viability and barrier integrity [44]. Our findings showed that co-treatment with the optimized monomer combination significantly rescued these antioxidant enzymes and suppressed MDA accumulation. This robust antioxidant effect is likely driven by the free-radical-scavenging capacities of Forsythoside A and Esculin, which work in tandem with the cytoprotective properties of Eleutheroside E and Anemoside B4 [45,46,47].

Third, we demonstrated that the optimized combination protects and restores the physical cellular barrier. The intestinal epithelial mechanical barrier is maintained by tight junction protein complexes, primarily ZO-1, Claudin-1, and Occludin. These proteins seal the paracellular space and regulate mucosal permeability. When these tight junctions are disrupted—as observed under 7S globulin exposure—paracellular permeability increases, allowing luminal antigens and pathogens to penetrate the mucosa and enter systemic circulation, which worsens enteritis [48,49]. In this study, the optimized monomer combination significantly upregulated the transcription of ZO-1, Claudin-1, and Occludin in 7S globulin-treated cells, promoting the repair and stabilization of the epithelial junctional complex.

At the molecular level, these systemic protective actions are mediated through the suppression of the NF-κB/MAPK signaling cascade [31,50,51,52]. The NF-κB and MAPK pathways are central regulators of mammalian inflammatory networks, cell survival, and barrier functions. MAPK is organized into three downstream cascades: ERK, p38 MAPK, and JNK. JNK and p38 MAPK are activated by physical and chemical stresses, including soy allergens. Upon phosphorylation, these MAPKs stimulate downstream transcription factor NF-κB p65 [4,53]. NF-κB p65 then translocates to the nucleus, where it drives the transcription of pro-inflammatory cytokines, including TNF-α, IL-1β, and IL-6. Furthermore, elevated TNF-α stimulates the expression of iNOS, which catalyzes the production of excessive nitric oxide (NO). This accumulation of NO further activates NF-κB, creating a self-amplifying feed-forward loop that sustains chronic mucosal damage and exacerbates oxidative stress [54]. In parallel, activation of the p38 MAPK pathway generates massive amounts of intracellular ROS, which damages mitochondria, disrupts tight junctions, and triggers lipid peroxidation [55].

To contextualize our findings, the JNK and p38 MAPK cascades are known to be key regulators of tight junction (TJ) remodeling under stress. In jejunal IPEC-J2 cells, selective activation of p38 MAPK and JNK directly triggers the dynamin-dependent endocytosis and lysosomal degradation of core tight junction proteins (TJPs, such as claudins and ZO-1) under both toxicological stress and nutrient starvation [56]. In our study, although TJ preservation was evaluated at the transcript (mRNA) level, the significant downregulation of total JNK and p38 MAPK proteins by Combination VI suggests indicate a blockade of these downstream, phosphorylation-driven endocytic degradation cascades. By inhibiting JNK and p38 activation, our optimized formulation may contribute to maintaining tight-junction-related gene expression, thereby maintaining the physical barrier integrity of porcine intestinal epithelial cells under 7S globulin challenge [57].

Our findings showed that 7S globulin exposure increased the mRNA and total protein expression of key NF-κB/MAPK pathway-related markers., as evidenced by significant increases in both the gene transcription and protein expression of p38 MAPK, JNK, NF-κB p65, and iNOS. However, treatment with the optimized monomer combination significantly suppressed these elevations at both the mRNA and protein levels, reducing the expression of pathway-related markers. By inhibiting the activation of p38 MAPK and JNK, the monomeric formulation prevents the nuclear translocation of NF-κB p65. This downregulates the downstream expression of pro-inflammatory cytokines and iNOS, breaking the self-amplifying feedback loop and reducing cellular injury. Concurrently, suppressing p38 MAPK reduces ROS production, preserving mitochondrial function and preventing oxidative damage to tight junction proteins.

In summary, this study demonstrates that combining these four traditional Chinese medicine monomers provides comprehensive, multi-target protection against soybean allergen-induced IPEC-J2 cell damage. The formulation simultaneously addresses the inflammatory cascade, oxidative damage, and physical barrier breakdown through the targeted inhibition of the NF-κB/MAPK pathway.

Despite these findings, several limitations should be acknowledged. First, this study was conducted only in an in vitro IPEC-J2 cell injury model; therefore, further validation in animal experiments, such as trials in weaned piglets, is required before practical application. Second, although the baseline concentrations of the four monomers were supported by previous single-factor screening studies from our research group, single-monomer and pairwise-combination controls were not included in the present combination-validation experiments. Thus, true synergistic interactions among the four monomers could not be quantitatively confirmed using a combination index or other interaction models. Third, although previous studies and the present Cell Counting Kit-8 assay provided preliminary evidence for the selected concentration ranges, the maximum tolerated concentration, long-term cytotoxicity, and safe application range of the optimized formulation require further evaluation. In addition, phosphorylated p65, p38 MAPK, and JNK were not detected; therefore, the activation status of the NF-κB/MAPK pathway could not be directly verified. The protective effects on epithelial barrier integrity were also evaluated mainly based on tight-junction-related mRNA expression, without transepithelial electrical resistance, permeability assays, or tight junction protein-level validation. Future studies should include in vivo validation, single-compound and combination-control groups, phosphorylation analysis, functional barrier assays, and protein-level detection of ZO-1, Claudin-1, and Occludin to further confirm the protective mechanism. Therefore, the current findings should be interpreted as preliminary in vitro evidence supporting the protective potential of the optimized monomer combination.

## 5. Conclusions

In conclusion, the optimized combination of traditional Chinese medicine monomers (50 mg/L Eleutheroside E, 25 mg/L Anemoside B4, 80 mg/L Forsythoside A, and 20 mg/L Esculin) significantly alleviated soybean 7S globulin-induced cell death, acute inflammatory damage, oxidative stress, and mechanical barrier damage in IPEC-J2 cells in vitro. These optimized combinatorial formulations are primarily mediated by modulating NF-κB/MAPK pathway-related markers, thereby downregulating downstream inflammatory gene expression and upregulating cellular tight junctions. These findings demonstrate that this optimized natural phytochemical blend effectively mitigates dietary-antigen-induced enteropathy model challenges in vitro, suggesting its potential as a promising candidate for developing green, antibiotic-free nutritional strategies to prevent post-weaning diarrhea in piglets.

## Figures and Tables

**Table 1:** Factors and levels of orthogonal test L_9_ (3^4^).

	Factors	Eleutheroside E mg/L	Anemoside B4 mg/L	Forsythoside A mg/L	Esculin mg/L
Levels					
1		100	100	80	40
2		50	50	40	20
3		25	25	20	10

**Table 2:** Orthogonal test group design.

Groups	Horizontal Combinations	Eleuthero Glycoside E (A) mg/L	Anemoside B4 (B) mg/L	Forsythoside A (C) mg/L	Esculin (D) mg/L
Control	/	0	0	0	0
I	A1B1C1D1	100	100	80	40
II	A1B2C2D2	100	50	40	20
III	A1B3C3D3	100	25	20	10
IV	A2B1C2D3	50	100	40	10
V	A2B2C3D1	50	50	20	40
VI	A2B3C1D2	50	25	80	20
VII	A3B1C3D2	25	100	20	20
VIII	A3B2C1D3	25	50	80	10
IX	A3B3C2D1	25	25	40	40

**Table 3:** Primer sequences.

Gene Name	Primer Sequence (5′-3′)
*ZO-1*	(F) CCAGGGAGAGAAGTGCCAGTAGG
	(R) TTTGGTGGGTTTGGTGGGTTGAC
*Claudin-1*	(F) CCATCGTCAGCACCGCACTG
	(R) CGACACGCAGGACATCCACAG
*Occludin*	(F) TGGCTGCCTTCTGCTTCATTGC
	(R) GAACACCATCACACCCAGGATAGC
*NF-κB p65*	(F) CTCGCTCGCACTCTTGCTCTC
* *	(R) AATAGGCGGACGAGGCAGAAG
*P38 MAPK*	(F) AAGACTCGTTGGAACCCCAG
* *	(R) TCCAGCAAGTCAACAGCCAA
*JNK*	(F) CAAGCACCTTCACTCTGCTG
* *	(R) TCAGGTGCTCTGTAGTAGCG
*iNOS*	(F) CAGCCCCACTATTCTCTGGAGGTA
* *	(R) CCTGGGAGGAACTAATGGAATA
*β-actin*	(F) CTGGACTTCGAGCAGGAGATGG (R) TTCGTGGATGCCGCAGGATTC

**Table 4:** Range analysis of the L_9_(3^4^) orthogonal test.

Factor	K_1_	K_2_	K_3_	R	Optimal Level
Eleuthero glycoside E (A) mg/L	109.22	111.61	109.70	2.39	A2
Anemoside B4 (B) mg/L	109.15	110.18	111.20	2.05	B3
Forsythoside A (C) mg/L	119.13	107.79	103.62	15.51	C1
Esculin (D) mg/L	110.04	111.13	109.36	1.78	D2

Note: K_i_ represents the average cell viability under that level. The range R = K_max_ − K_min_ indicates the importance of each monomer on viability.

## Data Availability

The original contributions presented in this study are included in the article/Supplementary Materials. Further inquiries can be directed to the corresponding authors.

## Supplementary Materials

The following supporting information can be downloaded at: https://www.mdpi.com/article/10.3390/vetsci13080758/s1, Figure S1: Original, uncropped immunoblot scan of β-actin (42 kDa); Figure S2: Original, uncropped immunoblot scan of JNK (48 + 54 kDa); Figure S3: Original, uncropped immunoblot scan of NF-κB p65 (65 kDa); Figure S4: Original, uncropped immunoblot scan of p38 (38–42 kDa); Figure S5: Original, uncropped immunoblot scan of INOS (130 kDa); Table S1: Densitometric quantification of Western blot bands for NF-κB/MAPK pathway-related proteins in IPEC-J2 cells exposed to soybean 7S globulin.
