# Supplementary material for: The Combination of Chinese Medicine Monomers Reduces 7S Globulin-Induced Damage to IPEC-J2 Cells In Vitro by Modulating NF-κB/MAPK Pathway-Related Markers
Source: Vet Sci. 2026 Jul 30;13(8):758. doi: 10.3390/vetsci13080758 (PMC13517924; doi:10.3390/vetsci13080758)
Supplement: Supplementary file 1 [file vetsci-13-00758-s001.zip › Supplementary_File_S1_Uncropped_Western_blot_images.pdf]

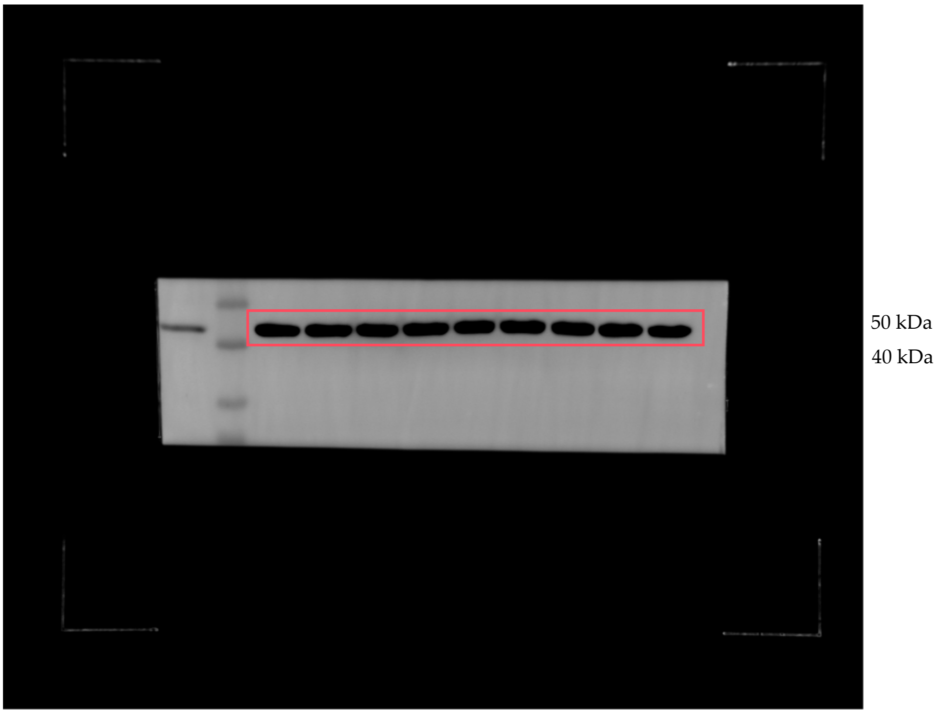

Figures S1. Original, uncropped immunoblot scan of β-actin (42 kDa)

Note: The nitrocellulose membrane was horizontally cut according to the prestained molecular weight markers prior to primary antibody incubation. The red dashed box outlines the cropped area presented in Figure 8. Lanes correspond to the following experimental groups: Lane 1-3: Control; Lane 4-6: 7S Model; Lane 7-9: 7S + Combination VI. The same as below.

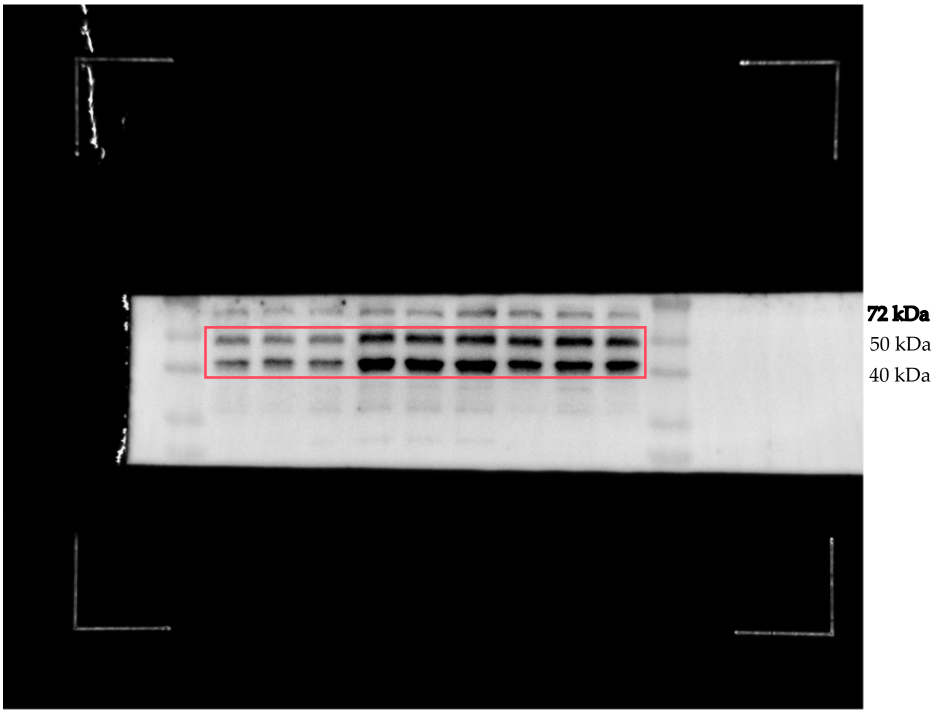

Figures S2. Original, uncropped immunoblot scan of JNK (48 + 54 kDa)

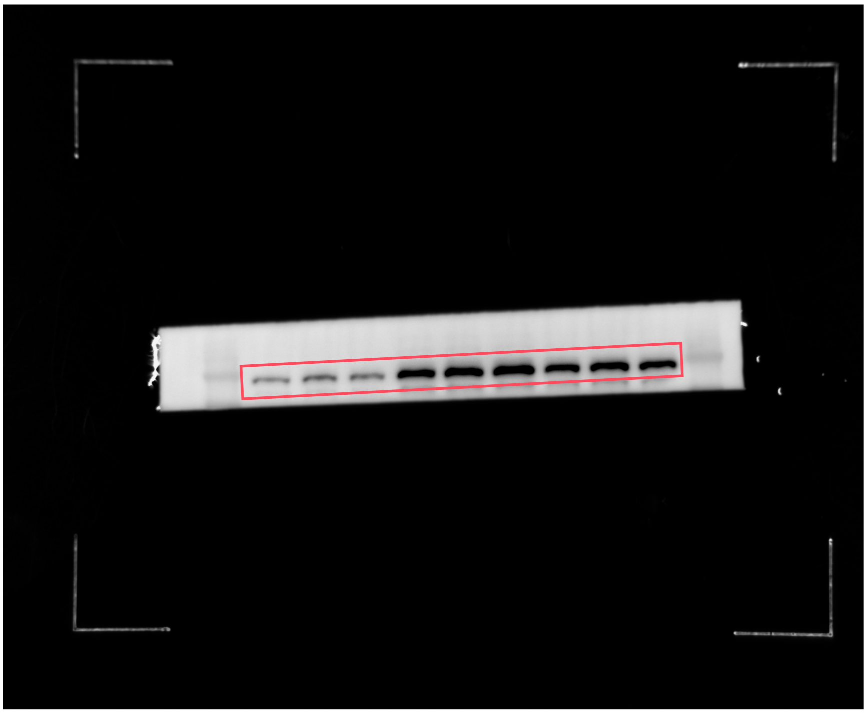

Figures S3. Original, uncropped immunoblot scan of NF-κB p65 (65 kDa)

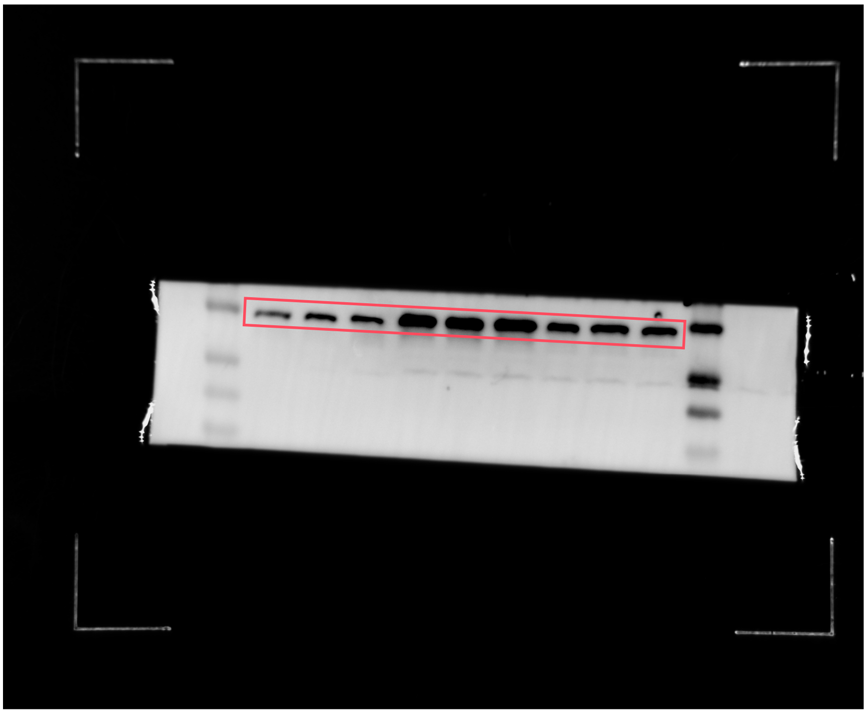

Figures S4. Original, uncropped immunoblot scan of p38 (38-42 kDa)

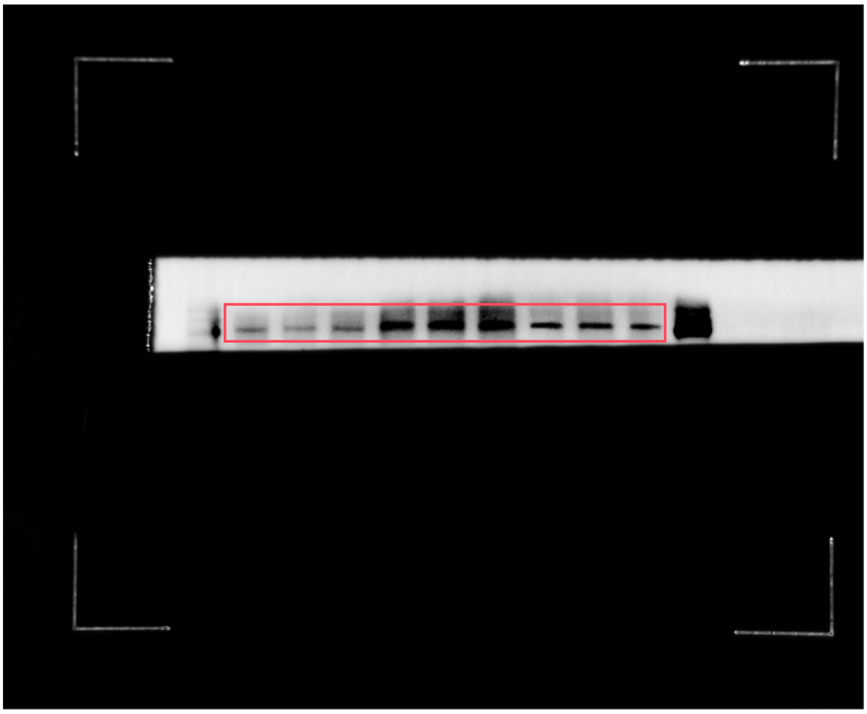

Figures S5. Original, uncropped immunoblot scan of INOS (130 kDa)
